# When digital engagement meets climate concern: hopelessness as an indirect link between digital addiction and eco-anxiety in adolescents

**DOI:** 10.3389/fpubh.2026.1830212

**Published:** 2026-06-17

**Authors:** Fatih Cebeci, Osman Akay, Selda Meydan, Sinem Arslankoç, Gizem Burcu Bolat, Deniz Say Şahin, Taner Artan

**Affiliations:** 1 Department of Social Work, Faculty of Health Sciences, Istanbul Medipol University, Istanbul, Türkiye; 2 UNEC Social Work and Social Innovations Research Center, Azerbaijan State University of Economics, Baku, Azerbaijan; 3 Department of Social Work, School of Health Sciences, Istanbul Medipol University, Istanbul, Türkiye; 4 Department of Social Work, Institute of Graduate Studies, Istanbul University-Cerrahpasa, Istanbul, Türkiye; 5 Department of Social Work, Faculty of Economics and Administrative Sciences, Burdur Mehmet Akif Ersoy University, Burdur, Türkiye; 6 Department of Social Work, Faculty of Health Sciences, Istanbul University-Cerrahpasa, Istanbul, Türkiye

**Keywords:** adolescents, digital addiction, eco-anxiety, hopelessness, social media exposure

## Abstract

Adolescents are increasingly exposed to climate-related content in digital environments; however, the psychological mechanisms underlying eco-anxiety in the context of problematic digital engagement remain insufficiently understood. This study examined the association between digital addiction and eco-anxiety and explored the mediating role of hopelessness in this relationship. The sample consisted of 3,561 high school students in Türkiye (56.4% female). Participants completed the Digital Addiction Scale for Teenagers, the Eco-Anxiety Scale–High School Form, and the Beck Hopelessness Scale. Associations among variables were examined using correlation analysis, and the mediating role of hopelessness was tested using a regression-based approach with bootstrapping. Digital addiction was weakly associated with eco-anxiety but showed a stronger association with hopelessness, while hopelessness was positively related to eco-anxiety. In the mediation analysis, digital addiction was associated with higher hopelessness, and hopelessness was associated with higher eco-anxiety. When hopelessness was included in the model, the direct effect of digital addiction on eco-anxiety became non-significant, while the indirect effect remained significant, indicating full mediation. These findings suggest that digital addiction may contribute to eco-anxiety primarily by increasing future-oriented pessimism and a diminished sense of control among adolescents. Eco-anxiety also differed across several socio-demographic and digital exposure variables. From a conceptual perspective, eco-anxiety appears to emerge not only as a response to environmental threat awareness but also as a psychological experience shaped by cognitive and emotional vulnerabilities within digital contexts. However, the cross-sectional design of the study limits causal interpretations of the findings. Addressing future-oriented pessimism and perceived inefficacy, alongside strengthening digital media literacy and climate-related coping resources, may support adolescent psychological wellbeing in digitally mediated environments.

## Introduction

With the rapid penetration of digital technologies into everyday life, adolescence has become a critical developmental stage characterized by intensified online interaction and heightened psychosocial vulnerabilities. The increasing use of social media and smartphones exposes adolescents to digital content for prolonged periods, often without adequate supervision; such exposure has been associated with addiction-like patterns marked by loss of control and impaired functioning. The World Health Organization Regional Office for Europe reports that problematic social media use among adolescents increased from 7% in 2018 to 11% in 2022, highlighting this trend as a significant risk factor for mental health (1). In Türkiye, problematic internet use has been reported in approximately 18.5% of high school students (2), and national survey data indicate that social media addiction scores are particularly elevated among individuals aged 14–18 (3). Problematic social media use is defined not merely by excessive duration of use but as a behavioral pattern characterized by diminished daily functioning, loss of control, and obsession-like symptoms (4). In addition to its behavioral characteristics, problematic digital use is also associated with prolonged daily engagement. Adolescents today spend a substantial amount of time online, often exceeding several hours per day (1) which increases their exposure to emotionally salient and potentially distressing content. Although problematic use cannot be defined solely by duration, extended and repetitive digital engagement may intensify psychological vulnerability by amplifying exposure to negative information and reducing opportunities for offline coping and regulation. Given that adolescents' cognitive and emotional regulation capacities are still developing, this age group is especially vulnerable to the addiction-like properties of digital technologies. Indeed, adolescents with problematic smartphone use have been found to exhibit higher levels of anxiety and suicidal ideation, underscoring the adverse effects of digital addiction on adolescent mental health (5).

Adolescence is widely regarded as a multidimensional developmental period during which cognitive capacity, personality structure, and social interaction skills undergo substantial reorganization (6, 7). During this stage, individuals encounter situations that demand intense adaptation shaped by both internal developmental dynamics and external expectations; such conditions may facilitate the emergence of rapid and less controlled behavioral patterns, thereby increasing the propensity for addictive behaviors (8). With the growing integration of digital technologies into daily life, digital addiction has been identified as one of the increasingly prevalent forms of addiction among adolescents (9, 10). Digital addiction is defined as a risk domain encompassing excessive, compulsive, and functionally impairing patterns of engagement with the internet, social media, smartphones, and other digital platforms, accompanied by symptoms of psychological dependence that negatively affect everyday functioning (11–14). This phenomenon is associated not only with the duration of use but also with the nature of the content to which individuals are exposed. Frequent exposure to themes such as climate crisis, environmental degradation, and global catastrophe in social media and digital news streams may heighten stress and anxiety levels. This suggests that climate change should be understood not only as a physical threat but also as a psychological health concern (15, 16). Adolescents, who typically exhibit lower tolerance for uncertainty, may perceive such threats as unpredictable and uncontrollable risks; this perception can foster increasingly negative expectations about the future, intensify feelings of loss of control and helplessness, and ultimately contribute to elevated levels of hopelessness defined as the tendency to hold negative beliefs and expectations about oneself and the future (17, 18).

The mental health impacts of the climate crisis and environmental uncertainties are becoming increasingly visible. Eco-anxiety is regarded as a significant psychological risk, particularly for children and adolescents. Research indicates that climate change is associated with psychological outcomes such as anxiety, stress, and functional impairment within this age group, and that concerns about the climate crisis are widely experienced among young people (19). From a developmental perspective, threats related to climate change have been reported to negatively affect adolescents' emotion regulation and adaptive processes (20).

Digital media represents one of the primary contexts through which adolescents are intensively exposed often to pre-dominantly negative content to information about climate and environmental issues. A recent study found that increased frequency of social media use is significantly associated with climate anxiety as well as perceptions of climate-related doom and hopelessness (21). These findings suggest that hopelessness may function as a critical psychological mechanism in understanding the impact of digital addiction on eco-anxiety. Nevertheless, although references to children and youth have increased in IPCC reports, the psychosocial and mental health consequences of climate change for this age groups remain insufficiently addressed (22).

A review of the existing literature indicates that digital addiction, eco-anxiety, and hopelessness have largely been examined within independent conceptual frameworks. However, studies that comprehensively investigate these variables within a single mediation model particularly among adolescent populations remain scarce. While some research has linked digital media use to general psychological symptoms (4), the climate anxiety literature has pre-dominantly focused on perception and affect. Moreover, studies examining the relationship between media exposure and climate anxiety have been conducted primarily with adult samples (23). In this context, the limited number of studies exploring how the relationship between digital addiction and eco-anxiety is shaped through hopelessness points to a theoretically important and yet insufficiently clarified research gap.

From a theoretical perspective, the relationships examined in this study can be understood within the framework of ecological systems theory (24), which conceptualizes individual development as shaped by interactions between the individual and multiple environmental systems. This perspective emphasizes that development is influenced by the environments in which individuals spend their time, the relationships they establish within these contexts, and broader socio-cultural conditions, as well as the processes through which these influences are internalized (25). Recent studies have also adopted ecological and systems-oriented perspectives to examine complex mental health outcomes and the interplay between environmental factors and psychological processes (26–28). In contemporary contexts, digital environments constitute a significant component of adolescents' immediate ecological context, while global issues such as climate change represent broader environmental influences. Within this framework, repeated exposure to digitally mediated, uncertainty-inducing content may contribute to the development of pessimistic future expectations and a diminished sense of control. In this process, hopelessness can be understood as a key mechanism linking digital exposure to emotional outcomes such as eco-anxiety. Accordingly, the present study adopts an ecological perspective to examine how digital addiction may be associated with eco-anxiety through the mediating role of hopelessness.

The present study aims to examine the impact of digital addiction on eco-anxiety among adolescents and to investigate the mediating role of hopelessness in this relationship. Specifically, it seeks to clarify how problematic engagement with digital technologies may influence climate-related anxiety both directly and indirectly. In doing so, the study provides a mechanism based empirical contribution to the literature. By adopting this perspective, eco-anxiety is conceptualized not merely as a response to environmental threat awareness but as a psychological experience emerging from the interaction between the cognitive emotional vulnerabilities shaped by the digital age and adolescents' perceptions of the future.

## Methods

### Research model

This study employed a cross-sectional quantitative research design to examine the effect of digital addiction on eco-anxiety among adolescents and to test the mediating role of hopelessness in this relationship. Quantitative research encompasses issues related to research design, measurement, and sampling, and emphasizes the detailed planning of data collection and analysis procedures prior to implementation (29).

A correlational and predictive research model was used to determine the relationships among variables and their predictive effects. Within the scope of the study, digital addiction was treated as the independent variable, eco-anxiety as the dependent variable, and hopelessness as the mediator.

### Participants

The sample consisted of 3,561 adolescents from high schools in Türkiye who voluntarily agreed to participate in the study and were selected using a convenience sampling method. The use of convenience sampling was based on accessibility and feasibility within school settings (30). Although this approach may limit the generalizability of the findings, efforts were made to include participants from different school types and socioeconomic backgrounds to enhance sample diversity. Therefore, the sample reflects a range of adolescent experiences within the study context, although it cannot be considered fully representative of the broader population. Of the participants, 56.4% were female (*n* = 2,008) and 43.6% were male (*n* = 1,553). The age distribution was pre-dominantly concentrated between 15 and 17 years, with the highest proportion being 16-year-olds (29.8%, *n* = 1,060).

In terms of grade level, a substantial proportion of the participants were enrolled in the 10th grade (27.2%, *n* = 968) and the 12th grade (29.1%, *n* = 1,036). Nearly half of the sample attended Anatolian High Schools (46.4%, *n* = 1,654), followed by Vocational High Schools (25.2%, *n* = 895) and Science High Schools (10.3%, *n* = 366). Monthly family income was primarily concentrated between 25,000–44,999 TL (31.4%, *n* = 1,118) and 45,000–64,999 TL (28.5%, *n* = 1,016). Regarding parental education, mothers most commonly had secondary school (24.6%, *n* = 876) or high school education (27.6%, *n* = 984), whereas fathers pre-dominantly held high school (33.4%, *n* = 1,188) or secondary school diplomas (23.6%, *n* = 842).

### Data collection tools

#### Sociodemographic information form

The Sociodemographic Information Form was developed by the researchers to obtain descriptive and contextual information about the participants. The selection of variables (e.g., age, sex, grade level, school type, family income, and parental education) was informed by a review of the relevant literature, particularly studies focusing on adolescent mental health, digital behavior, and environmental attitudes. These variables have been consistently identified as important contextual factors associated with psychological outcomes in adolescents. As the form was not designed as a psychometric measurement tool but rather as a descriptive data collection instrument, no validity or reliability analysis was conducted.

#### Digital Addiction Scale for Teenagers

The Digital Addiction Scale for Teenagers was developed by Seema et al. (31) to objectively assess adolescents' levels of digital addiction, and its Turkish validity and reliability were established by Ekşi and Yüksel (12). The scale employs a 7-point Likert format ranging from 1 (never) to 7 (very frequently). The instrument has a unidimensional structure consisting of 10 items. A sample item is “I feel bored when I cannot use my digital device.” Total scores are calculated by summing all item responses, yielding a possible range of 10 to 70, with higher scores indicating greater levels of digital addiction. Total scores are calculated by summing item responses, and no reverse-coded items are included. Higher scores indicate greater levels of digital addiction. The Cronbach's alpha coefficient was reported as 0.87 in the Turkish adaptation study, while it was calculated as 0.92 for the present sample.

#### Eco-Anxiety Scale–High School Form

The original scale was developed by Hogg et al. (32) to measure eco-anxiety in individuals aged 18 and over, and its Turkish validity and reliability were established by Uzun et al. (33). Given the absence of a measurement tool specifically targeting adolescent groups more affected by eco-anxiety, the high school form adapted into Turkish was developed by Erzen and Taşdemir (34). The scale consists of 10 items rated on a 4-point Likert scale ranging from 0 (never) to 3 (almost always) and comprises three subdimensions: emotional symptoms (Items 1–4), anxiety about personal impact (Items 5–7), and repetitive thinking (Items 8–10). The scale includes items such as “feeling nervous, anxious, or on edge” and “being unable to stop or control worrying” when thinking about climate change and environmental problems. Total scores are obtained by summing item responses, resulting in a possible range of 0 to 30, with higher scores indicating greater eco-anxiety. No reverse-coded items are included. The Cronbach's alpha coefficient was reported as 0.84 for the high school form and was calculated as 0.87 in the present study.

#### Beck Hopelessness Scale

The Beck Hopelessness Scale was developed by Beck et al. (17), and its Turkish validity and reliability study was conducted by Seber et al. (35). The scale consists of 20 true–false statements, with 11 keyed as “true” and 9 as “false,” and is designed for self-administration. A sample item is “My future seems dark to me.” Total scores are calculated by summing item scores, yielding a possible range from 0 to 20, with higher scores indicating greater hopelessness. Each response consistent with the scoring key is assigned 1 point, while inconsistent responses receive 0 points. The sum of these scores represents the hopelessness score, ranging from 0 to 20. Items 1, 6, 13, 15, and 19 assess feelings about the future; Items 2, 3, 9, 11, 12, 16, 17, and 20 measure loss of motivation; and Items 4, 7, 8, 14, and 18 evaluate expectations regarding the future. The Cronbach's alpha coefficient was reported as 0.86 in the Turkish adaptation study and calculated as 0.84 in this study.

### Data collection process

Data were collected using a set of instruments that included the Sociodemographic Information Form and the measurement scales employed in the study. Following approval from the ethics committee, data were gathered through face-to-face administration in secondary education institutions between November 2025 and January 2026. Prior to data collection, participants were informed about the purpose of the study, and informed consent was obtained from the participants. Parental consent was obtained from the parents or legal guardians of the participants prior to participation. Participation was voluntary, and students were informed that they could withdraw from the study at any time without any consequences. Confidentiality and anonymity were assured throughout the research process. A total of 3,593 questionnaires were initially collected. During the data screening process, forms that were incomplete, improperly completed, or contained inconsistent response patterns were excluded. Following this procedure, the final analytic sample consisted of 3,561 adolescents with complete and valid data, and all analyses were conducted using this dataset.

### Data analysis

Statistical analyses were conducted using IBM SPSS Statistics 25.0 and the PROCESS Macro (v4.2). Normality assumptions were evaluated using skewness and kurtosis values, which indicated no substantial deviation from normality (36). Internal consistency of the measurement tools was assessed using Cronbach's alpha coefficients. Descriptive statistics, including frequencies, percentages, means, and standard deviations, were calculated to summarize participant characteristics and scale scores. Independent samples *t*-tests and one-way ANOVA were performed to examine differences in eco-anxiety across demographic variables. Tukey HSD and Games–Howell tests were used for *post-hoc* comparisons depending on the homogeneity of variances. Pearson correlation analysis was conducted to explore associations among the study variables, and correlation coefficients were interpreted based on Cohen's (37) guidelines.

The mediating role of hopelessness in the relationship between digital addiction and eco-anxiety was tested using Hayes' (38) PROCESS Macro (Model 4). Sex, age, family income, and parental education levels were included as covariates to control for potential confounding effects. The significance of indirect effects was evaluated using bootstrap confidence intervals based on 5,000 resamples; confidence intervals that did not include zero were considered statistically significant. The level of statistical significance was set at *p* < 0.05. After excluding cases with missing or invalid responses during data screening, all analyses were conducted using a complete-case approach. No missing data imputation procedure was applied. As no universally established or clinically validated cutoff scores are available for the measures used in this study, all variables were analyzed as continuous constructs.

## Results

The results of the descriptive analyses, correlation tests, and the mediation model are presented in this section, beginning with an overview of participants' digital habits and environmental orientations.

As shown in Table 1, 90.8% of participants reported using the internet for at least 3 h per day. Exposure to climate- and environment-related content was most commonly reported as “occasionally” (41.4%). The most frequently reported emotional response to such content was sadness (34.5%), followed by anxiety (24.0%). Participants most often reported a moderate level of personal responsibility regarding environmental issues (43.2%), and the majority described themselves as partially knowledgeable about environmental topics (58.1%). These variables were included to provide descriptive context for adolescents' digital habits and environmental orientations.

**Table 1:** Participants' internet use habits and attitudes toward environmental issues.

Variable	Category	Frequency (*N*)	Percentage (%)
Average daily internet use	0–2 h	328	9.2
	3–5 h	1,376	38.6
	6–8 h	1,371	38.5
	9 h or more	486	13.6
Exposure to climate and environmental content on social media	Never	416	11.7
	Rarely	977	27.5
	Occasionally	1,474	41.4
	Frequently	552	15.5
	Every day	142	4.0
Emotional responses to climate-related content	Anxiety	853	24.0
	Fear	594	16.7
	Sadness	1,228	34.5
	Guilt	246	6.9
	Motivation	94	2.6
	Hope	58	1.6
	Indifference	488	13.7
Sense of personal responsibility regarding environmental issues	None	324	9.1
	Low	1,095	30.8
	Moderate	1,538	43.2
	High	490	13.8
	Very high	114	3.2
Perceived environmental knowledge	No	563	15.8
	Partially	2,070	58.1
	Yes	928	26.1
Total		3,561	100

Table 2 presents the results of the independent samples *t*-test and one-way analysis of variance (ANOVA) conducted to examine whether eco-anxiety scores differed across participants' sociodemographic characteristics. The independent samples *t*-test revealed a statistically significant difference in eco-anxiety scores by sex (*t* = 2.484, *p* < 0.05), with female students reporting higher mean scores (*M* = 9.84, *SD* = 5.67) than male students (*M* = 9.34, *SD* = 6.22). No statistically significant differences were observed across age groups (*F* = 1.381, *p* > 0.05). Eco-anxiety scores differed significantly by school type (*F* = 4.728, *p* < 0.001). Games–Howell *post hoc* comparisons indicated that students attending Science High Schools reported higher eco-anxiety than those enrolled in Anatolian and Vocational High Schools. Additionally, Imam Hatip High School students demonstrated higher eco-anxiety levels compared to Vocational High School students. A statistically significant difference was also found across income levels (*F* = 5.464, *p* < 0.001). *Post hoc* analyses showed that participants in the lowest income group (0–24,999 TL) reported higher eco-anxiety scores than those in the middle-income categories (25,000–84,999 TL). Although the differences in eco-anxiety across sex, school type, and income level were statistically significant, the calculated effect sizes were small (Cohen's *d* = 0.084 for sex; η^2^ = 0.007 for school type; and η^2^ = 0.008 for income level).

**Table 2:** Comparison of eco-anxiety levels by participants' sociodemographic characteristics.

Variables	Groups	*N*	*M*	*SD*	Test statistic (*t*/*F*)	*p*	Effect size (*d*/η^2^)	*Post-Hoc* difference
Sex	Female	2,008	9.84	5.67	2.484	0.013^*^	0.084	Female> male
	Male	1,553	9.34	6.22				
Age	14 and under	234	9.00	5.59	1.381	0.228	—	—
	15	822	9.80	6.11				
	16	1,060	9.60	5.78				
	17	885	9.61	5.96				
	18	412	9.41	5.95				
	19 and above	148	10.47	6.11				
School type	Anatolian high school	1,654	9.45	5.82	4.728	0.000^***^	0.007	Science >Anatolian, vocational Imam Hatip >vocational (games–Howell)
	Science high school	366	10.62	5.40				
	Vocational high school	895	9.14	6.45				
	Imam Hatip high school	278	10.34	5.80				
	Private high school	220	9.96	5.64				
	Social sciences high school	148	10.11	5.19				
Income level	0–24,999 TL	298	11.01	6.31	5.464	0.000^***^	0.008	0–24 k >25–44 k 0–24 k >45–64 k 0–24 k >65–84 k (games–Howell)
	25,000–44,999 TL	1,118	9.10	6.14				
	45,000–64,999 TL	1,016	9.57	5.80				
	65,000–84,999 TL	521	9.72	5.54				
	85,000–104,999 TL	402	10.00	5.67				
	105,000 TL and above	206	9.67	5.88				

*N* = 3,561.

*M*, mean; SD, standard deviation.

^*^p < 0.05, ^**^p < 0.01, ^***^p < 0.001.

Table 3 presents the results of the one-way analysis of variance (ANOVA) conducted to examine whether eco-anxiety scores differed according to participants' internet use habits and environmental attitudes. A statistically significant difference was found across daily internet usage groups (*F* = 3.944, *p* < 0.01). Tukey *post hoc* comparisons indicated that participants who reported using the internet for 9 h and above had higher eco-anxiety scores than those using the internet for 3–5 h. Eco-anxiety scores differed significantly according to exposure to climate and environmental content on social media (*F* = 91.425, *p* < 0.001). Games–Howell *post hoc* comparisons showed that participants with more frequent exposure reported higher eco-anxiety scores than those with lower exposure levels. A statistically significant difference was also observed across levels of personal responsibility regarding environmental issues (*F* = 28.141, *p* < 0.001). Participants reporting high and very high levels of responsibility demonstrated higher eco-anxiety scores compared to those reporting moderate or low levels. Similarly, eco-anxiety scores varied significantly based on perceived environmental knowledge (*F* = 14.459, *p* < 0.001). Participants who described themselves as knowledgeable or partially knowledgeable reported higher eco-anxiety than those indicating no environmental knowledge. Effect size analyses revealed that exposure to climate and environmental content on social media had a moderate effect on eco-anxiety (η^2^ = 0.093), while sense of personal responsibility had a small effect (η^2^ = 0.031). The effects of daily internet use and perceived environmental knowledge were very small (η^2^ = 0.003 and η^2^ = 0.008, respectively).

**Table 3:** Comparison of eco-anxiety levels according to participants' internet use habits and environmental.

Variables	Groups	*N*	*M*	*SD*	Test statistic (*F*)	*p*	Effect size (*d*/η^2^)	*Post-Hoc* difference
Average daily internet use	0–2 h	328	9.87	6.05	3.944	0.008^**^	0.003	9 h and above >3–5 h (Tukey)
	3–5 h	1,376	9.34	5.80				
	6–8 h	1,371	9.58	5.90				
	9 h and above	486	10.38	6.20				
Exposure to climate and environmental content on social media	Never	416	6.55	6.73	91.425	0.000^***^	0.093	Every day >frequently >occasionally >rarely >never (Games–Howell)
	Rarely	977	8.29	5.67				
	Occasionally	1,474	10.10	5.58				
	Frequently	552	11.79	4.90				
	Every day	142	14.28	5.30				
Sense of personal responsibility regarding environmental issues	None	324	8.57	7.20	28.141	0.000^***^	0.031	Very high, high >moderate >low (Games–Howell)
	Low	1,095	8.80	6.15				
	Moderate	1,538	9.60	5.49				
	High	490	11.53	4.87				
	Very high	114	12.49	6.66				
Perceived environmental knowledge	No	563	8.48	6.53	14.459	0.000^***^	0.008	Yes, partially >no (Games–Howell)
	Partially	2,070	9.69	5.60				
	Yes	928	10.16	6.15				

*N* = 3,561.

*M*, mean; SD, standard deviation.

^*^p < 0.05, ^**^p < 0.01, ^***^p < 0.001.

Table 4 presents the internal consistency coefficients of the measurement scales used in the study. Cronbach's alpha values were calculated as 0.875 for the Eco-Anxiety Scale–High School Form, 0.928 for the Digital Addiction Scale for Teenagers, and 0.849 for the Beck Hopelessness Scale.

**Table 4:** Internal consistency coefficients of the measurement scales.

Scale	Number of items	Cronbach's alpha
Eco-Anxiety Scale–high school form	10	0.875
Digital Addiction Scale for teenagers	10	0.928
Beck Hopelessness Scale	20	0.849

Table 5 presents the descriptive statistics and Pearson correlation coefficients among the study variables. Skewness and kurtosis values fell within the acceptable range (±2), indicating no substantial deviation from normality. Correlation analyses revealed a positive, small-to-moderate association between digital addiction and hopelessness (*r* = 0.252, *p* < 0.01). Hopelessness also demonstrated a positive, small correlation with eco-anxiety (*r* = 0.145, *p* < 0.01). Furthermore, the direct correlation between digital addiction and eco-anxiety was positive but very weak in magnitude (*r* = 0.040, *p* < 0.05).

**Table 5:** Descriptive statistics and correlation coefficients among study variables.

Variables	*M*	*SD*	Skewness	Kurtosis	1	2	3
1. Eco-anxiety	9.62	5.92	0.271	−0.364	—		
2. Digital addiction	42.80	14.39	−0.136	−0.804	0.040^*^	—	
3. Hopelessness	6.74	4.67	0.769	−0.019	0.145^**^	0.252^**^	—

*N* = 3,561.

^*^p < 0.05, ^**^p < 0.01.

**Figure 1:**
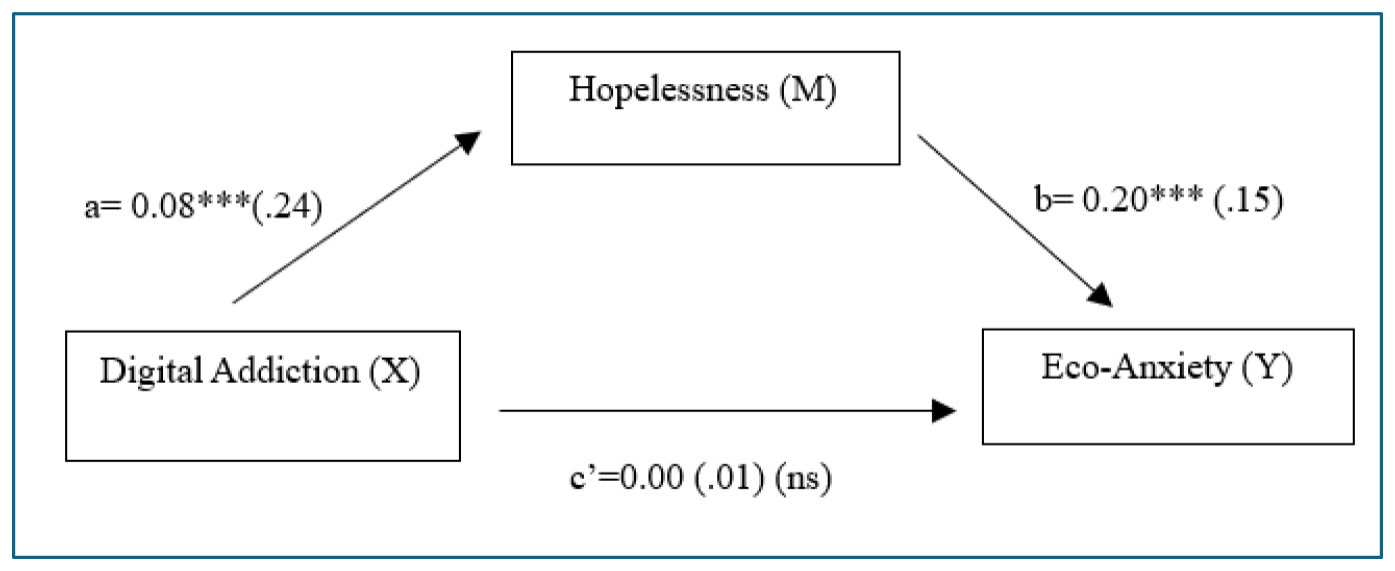
The mediating role of hopelessness in the relationship between digital addiction and eco-anxiety. Values represent unstandardized regression coefficients (*B*). Covariates (sex, age, family income, mother's and father's education levels) were controlled for in the model. Values in parentheses represent the standardized regression coefficients (β). ***p < 0.001, ns: non-significant.

The mediation analysis results are presented in Table 6, and the tested model is illustrated in Figure 1. After controlling for demographic variables (sex, age, family income, and parental education levels), digital addiction significantly predicted hopelessness (β = 0.24, *p* < 0.001), and hopelessness significantly predicted eco-anxiety (β = 0.15, *p* < 0.001). When hopelessness was included in the model, the direct effect of digital addiction on eco-anxiety became non-significant (β = 0.01, *p* > 0.05). The significance of the indirect effect was tested using a bootstrap procedure with 5,000 resamples, and the 95% confidence interval did not include zero [*CI* (0.027, 0.046)]. Given the non-significant direct effect alongside a highly significant indirect effect, this indicates a significant full mediation effect. Regarding the covariates, higher maternal education (β = −0.05, *p* < 0.05) and family income (β = −0.12, *p* < 0.001) were associated with lower hopelessness scores. Higher family income was also associated with higher eco-anxiety (β = 0.04, *p* < 0.05). In addition, age was positively associated with hopelessness (β = 0.03, *p* < 0.05), while female students reported higher eco-anxiety (β = −0.06, *p* < 0.001) and male students reported higher hopelessness (β = 0.09, *p* < 0.001).

**Table 6:** The mediating role of hopelessness in the relationship between digital addiction and eco-anxiety.

Predictors	Mediator model				Dependent variable model			
	(Outcome: hopelessness)				(Outcome: eco-anxiety)			
	B (β)	SE	*t*	95% CI	*B (β)*	SE	*t*	95% CI
Constant	3.72^***^	0.46	8.00	[2.80, 4.63]	9.10^***^	0.62	14.75	[7.89, 10.31]
Digital addiction (X)	0.08^***^ (0.24)	0.01	14.67	[0.07, 0.09]	0.00 (0.01)	0.01	0.31	[−0.01, 0.02]
Hopelessness (*M*)	—	—	—	—	0.20^***^ (0.15)	0.02	8.82	[0.15, 0.24]
Covariates								
Sex	0.89^***^ (0.09)	0.15	5.87	[0.59, 1.19]	−0.71^***^ (−0.06)	0.20	−3.53	[−1.10, −0.32]
Age	0.13^*^ (0.03)	0.06	2.12	[0.01, 0.25]	0.04 (0.01)	0.08	0.55	[−0.11, 0.20]
Family income	−0.41^***^ (−0.12)	0.06	−6.31	[−0.54, −0.28]	0.20^*^ (0.04)	0.09	2.33	[0.03, 0.37]
Mother's education level	−0.18^*^ (−0.05)	0.08	−2.18	[−0.34, −0.02]	−0.04 (−0.01)	0.11	−0.33	[−0.25, 0.18]
Father's education level	0.01 (0.00)	0.09	0.06	[0.17, 0.18]	−0.11 (−0.02)	0.12	−0.95	[−0.34, 0.12]
R^2^	0.09				0.03			
*F*	61.48^***^				13.61^***^			
Indirect effect								
Digital addiction → hopelessness → eco-anxiety	0.015^***^ (0.036)	0.002 (0.005)	—	[0.011, 0.019] ([0.027, 0.046])

*N* = 3,561.

Bootstrap sample size = 5,000; *B*, unstandardized regression coefficients; β, standardized regression coefficients; SE, standard error; *t*, t-test statistics; CI, confidence interval.

Sex was coded as 1 = Female, 2 = Male.

Family income and parental education levels were entered as continuous variables.

Values in parentheses represent the standardized regression coefficients (β).

^*^p < 0.05, ^**^p < 0.01, ^***^p < 0.001.

## Discussion

These findings can be interpreted within the framework of Ecological Systems Theory, which posits that adolescents' psychological outcomes are shaped through dynamic interactions between individual vulnerabilities and multiple environmental systems (24, 25), and has been increasingly applied to understand contemporary adolescent experiences in complex social contexts (39). In this context, digital environments may function as proximal ecological systems that intensify adolescents' exposure to climate-related threats and uncertainty, while hopelessness can be conceptualized as an internalized cognitive-emotional mechanism through which these environmental influences are processed by the individual. Within this framework, the present study demonstrates that the relationship between digital addiction and eco-anxiety in adolescents is better explained not through a direct and linear pathway, but through an indirect psychological process operating via hopelessness. The findings are consistent with contemporary perspectives that conceptualize eco-anxiety not merely as a response to environmental threat awareness, but as a multidimensional psychological experience shaped by the interplay of factors such as the rhythm of digital life, media exposure, socioeconomic vulnerabilities, and educational context (15, 19, 40, 41). In this regard, excessive and uncontrolled engagement in digital environments may contribute to eco-anxiety not by directly generating environmental concern, but by amplifying negative future expectations, feelings of helplessness, and diminished self-efficacy. Accordingly, eco-anxiety can be understood, based on the findings of this study, as an experience that emerges within a cognitive-emotional context characterized by heightened hopelessness.

### Socio-demographic vulnerabilities and psychological correlates

Sex findings indicated that female adolescents reported higher levels of eco-anxiety. Evidence from multinational youth samples similarly suggests that climate anxiety and future uncertainty tend to be more pronounced among females, pointing to a cross-cultural pattern (19). Studies linking eco-anxiety to the emotional and cognitive internalization of environmental threats also emphasize that females may experience such threats as more immediate and personally relevant (40). Developmental psychopathology literature further indicates that adolescent girls are generally more vulnerable to internalizing symptoms such as anxiety and hopelessness, particularly in the presence of future-oriented stressors (42).

Additionally, given that females are often reported to be more prone to social media addiction while males tend toward gaming addiction (43), greater exposure to climate-related catastrophic content through social media may contribute to elevated eco-anxiety. Therefore, the observed sex difference may reflect not only emotional variation but also developmental differences in processing future- and control-related cognitions.

School-type differences suggest that educational context and cognitive exposure such as access to scientific knowledge, critical awareness, and future-oriented scenarios may shape the intensity of environmental concerns. Higher eco-anxiety among Science High School students appears consistent with the view that increased exposure to scientific discourse on climate risks may heighten risk perception (44). Ojala's (45) hope and coping framework similarly highlights that cognitive internalization of environmental threats may foster anxiety when not balanced by perceived agency and sources of hope. The higher eco-anxiety observed among Imam Hatip High School students compared to Vocational High School students may indicate that contexts emphasizing values, responsibility, and moral obligation could intensify the personal relevance of environmental threats. This interpretation is consistent with literature suggesting that eco-anxiety is shaped not only by cognitive awareness but also by cultural and moral frameworks (19).

Income-related findings appear meaningful at two levels. First, higher eco-anxiety among lower-income adolescents may reflect greater vulnerability to climate-related risks, limited adaptive capacity, and the psychological consequences of environmental injustice (15, 46). This interpretation aligns with evidence indicating that eco-anxiety in youth should be examined alongside social inequalities (41). Second, mediation results also suggested that eco-anxiety may increase with higher income levels. This seemingly paradoxical pattern may be associated with greater access to information, increased media engagement, and heightened awareness of climate risks as long-term global threats (19). In this context, eco-anxiety may emerge through multiple pathways, including both structural vulnerability and heightened awareness, rather than along a single socioeconomic gradient (40, 45).

### Digital exposure and the dual role of social media

Findings related to digital habits indicate that greater internet use and more frequent exposure to climate-related content were associated with higher eco-anxiety. Similarly, stronger feelings of personal responsibility and higher perceived environmental knowledge corresponded with elevated eco-anxiety levels. These results are consistent with research suggesting that climate-related content particularly when framed through catastrophic narratives may generate feelings of helplessness and anxiety among young people (19, 47). Previous research suggests that repeated exposure to pre-dominantly negative online news may intensify psychological distress, particularly through cumulative emotional and cognitive strain associated with continuous engagement with distressing content (48, 49). Emerging studies focusing specifically on climate news also support this risk pathway (50). In this context, the findings suggest that patterns characteristic of digital addiction may contribute to eco-anxiety and hopelessness not only through increased screen time but also through prolonged and repeated exposure to pre-dominantly negative online content.

### The mediating role of hopelessness

The primary theoretical contribution of this study lies in empirically supporting an indirect pathway linking digital addiction to eco-anxiety through hopelessness. Preliminary analyses indicated that while the direct association between digital addiction and eco-anxiety was weak, digital addiction was significantly associated with hopelessness, which in turn predicted eco-anxiety. Mediation analysis further demonstrated that when hopelessness was included in the model, the direct relationship between digital addiction and eco-anxiety became non-significant, and the bootstrap confidence interval confirmed the significance of the indirect effect. Together, these findings support a full mediation pattern, suggesting that digital addiction may influence eco-anxiety primarily through its association with pessimistic future orientations and reduced perceived agency. This pattern is consistent with studies linking digital media use to hopelessness and psychological distress (51, 52) and with theoretical frameworks that position hope and agency as central components in understanding eco-anxiety (40, 45). Research emphasizing the centrality of helplessness in youths' climate-related psychological burden further strengthens the conceptual grounding of these findings (53, 54).

Building on these findings, the most important empirical contribution of this study lies in demonstrating that the relationship between digital addiction and eco-anxiety operates indirectly through hopelessness. While previous studies have examined digital media use and eco-anxiety separately, the present findings provide evidence for a psychological mechanism linking these constructs. The full mediation pattern observed suggests that digital addiction does not directly produce eco-anxiety; rather, it contributes to a pessimistic future orientation, which in turn increases eco-anxiety.

From a methodological perspective, this study contributes to the literature by employing a large adolescent sample and testing a mediation model using bootstrapping procedures. The use of PROCESS Macro allowed for a robust examination of indirect effects, strengthening the validity of the findings. Additionally, focusing on a non-Western adolescent population provides valuable context-specific insights into the relationship between digital addiction, hopelessness, and eco-anxiety. Finally, covariate results contribute to the ecological coherence of the model. Higher maternal education and family income were associated with lower hopelessness, supporting longstanding evidence that socioeconomic resources may foster psychological resilience and more positive future expectations (55). The positive association between age and hopelessness may reflect the increasing salience of future planning and uncertainty during later stages of adolescence. These findings point to a risk ecosystem shaped by digital exposure, socioeconomic resources, educational context, and developmental processes rather than a single dominant risk factor (15, 41).

The findings of this study have several important theoretical and practical implications. From a theoretical perspective, eco-anxiety should not be conceptualized solely as a response to environmental threat awareness but as a psychologically mediated process shaped by cognitive vulnerabilities such as hopelessness. By identifying hopelessness as a key mediating mechanism, the present study extends the literature by offering a mechanism-based explanation of how digital environments may influence eco-anxiety in adolescents. From a practical perspective, the findings suggest that interventions targeting adolescent mental health should move beyond approaches focused solely on reducing screen time or increasing environmental awareness. Instead, strategies that strengthen hope, perceived control, and self-efficacy may play a critical role in mitigating eco-anxiety in digitally mediated contexts.

## Limitations

Several limitations should be considered when interpreting these results. The cross-sectional design does not permit causal conclusions or clear temporal ordering among digital addiction, hopelessness, and eco-anxiety. Longitudinal studies would provide stronger evidence regarding the directionality of these relationships. All data were collected through self-report measures, which may introduce common method bias and socially desirable responding. Future research could benefit from incorporating multi-informant data or objective indicators of digital use. The use of convenience sampling and the focus on adolescents in Türkiye may limit the generalizability of the findings to other cultural contexts. Replication across diverse populations would strengthen the external validity of the proposed associations. Although several demographic variables were controlled for, other factors relevant to adolescent mental health such as depression, anxiety, emotion regulation, and perceived social support were not included and may have influenced the observed relationships. Finally, the direct association between digital addiction and eco-anxiety was small despite reaching statistical significance, suggesting that interpretations should focus primarily on the indirect pathway identified in the mediation model.

## Conclusion

This study contributes to the literature by examining the relationship between digital addiction and eco-anxiety in adolescents through the mediating role of hopelessness. The results indicate that digital addiction may be associated with eco-anxiety not through a direct pathway but through a process linked to weakened future expectations, reduced sense of control, and diminished self-efficacy. Eco-anxiety can therefore be understood not only as a response to environmental threat awareness but also as a multidimensional psychological experience shaped by cognitive and emotional vulnerabilities within an increasingly digitalized context.

Intensive exposure to climate-related content in digital media environments often framed through catastrophic narratives may reinforce future-oriented pessimism and feelings of inefficacy among adolescents. Eco-anxiety appears more likely to develop within such a cognitive framework. This interpretation aligns with theoretical approaches emphasizing the roles of hope, self-efficacy, and collective coping capacity in understanding psychological responses to climate change. Viewing climate anxiety solely as a pathological reaction may therefore be overly reductive; its functional impact becomes more apparent when accompanied by heightened hopelessness. The socio-demographic variations observed in the study further highlight the contextual nature of eco-anxiety. Higher levels among female adolescents, together with differences associated with school type and income level, suggest that eco-anxiety may emerge at the intersection of sex, educational context, and socioeconomic conditions. Environmental concerns may thus reflect not only individual sensitivity but also broader structural inequalities that shape perceptions of risk and future security.

From a practical perspective, the findings imply that interventions targeting adolescents may benefit from moving beyond strategies limited to reducing screen time or increasing environmental knowledge. Approaches that strengthen digital media literacy, enhance awareness of algorithmically driven content, support emotion regulation, and cultivate protective psychological resources particularly hope and future orientation are likely to be more responsive to the complexity of this phenomenon. School-based mental health programs, family-focused psychoeducation, and opportunities for constructive collective engagement with climate issues may serve protective functions against the convergence of hopelessness and eco-anxiety. The study offers a framework for understanding how large-scale processes such as digitalization and the climate crisis intersect with adolescent mental health through pathways linked to hopelessness. Longitudinal designs, qualitative inquiries, and cross-cultural comparisons would help clarify the temporal and contextual dynamics of the digital addiction–hopelessness eco-anxiety relationship and advance both theoretical and applied scholarship in this emerging field.

## Data Availability

The original contributions presented in the study are included in the article/supplementary material, further inquiries can be directed to the corresponding author.
